# Abdominal circumference and its relationship with the incidence of hypotension during elective cesarean section under spinal anesthesia at public hospitals of Addis Ababa, Ethiopia, 2024

**DOI:** 10.1371/journal.pone.0354843

**Published:** 2026-10-23

**Authors:** Wakjira Birhanu Kono, Gemechis Akuma Wayesa, Misrak Weldeyohannes Menchamo, Senait Awekea Yadeta, Gutu Leta Dinka

**Affiliations:** 1 Department of Anesthesia, Institute of Health Science, Wollega University, Nekemte, Ethiopia; 2 Department of Anesthesia, College of Health Science, Addis Ababa University, Addis Ababa, Ethiopia; University Hospital Knappschaftskrankenhaus Bochum, GERMANY

## Abstract

**Background:**

Hypotension is a common complication in pregnant mothers during cesarean section after spinal anesthesia. Identifying the association between abdominal circumference and the likelihood of developing hypotension following a cesarean delivery while under spinal anesthesia can facilitate preventing hypotension.

**Objectives:**

This study aims to assess the relationship between the abdominal circumference of pregnant women and the occurrence of hypotension during cesarean section under spinal anesthesia at public hospitals in Addis Ababa, Ethiopia, 2024.

**Method:**

A prospective observational study through a simple systematic random sampling technique was conducted on 360 pregnant mothers who underwent elective cesarean section under spinal anesthesia from January 7, 2024, to March 30, 2024. Data was collected using a data collection questionnaire and checklist. The data was entered using Epi Info 7.2 and exported into SPSS 26. The Patients were categorized by their median abdominal circumference, < 102 cm (small abdominal circumference (SAC)) and ≥102 cm (large abdominal circumference (LAC)). The chi-square test was used to compare the numerical and percentage representations of the categorical variables. Descriptive analyses, bivariate logistic regression, and multivariate regression were carried out. In multivariate variables, p ≤ 0.05 was used as statistical significance, and an odds ratio was used for the strength of association.

**Result:**

Among 360 study subjects, the incidence of post-spinal hypotension was 76.67% with 95%CI [71.9–80.9]. The Large abdominal circumference (LAC) group showed a greater maximal decrease in MAP from baseline compared to the SAC group (LAC 85.6% and SAC 63.3%, p < 0.001). The identified associated factors with the incidence of hypotension were LAC (AOR = 2.98, 95% CI [1.57, 5.65] p = 0.01); BMI > 25 kg/m2 (AOR = 2.3, 95%CI [1.16–4.6], P = 0.018); baby weight> 4 kg (AOR = 11.41 95%CI [2.0–64.86] P = 0.006); sensory block of above T6 level (AOR = 2.22, 95%CI [1.12, 4.42]) p = 0.023) and greater than 500 ml preload given (AOR = 0.42 [95%CI 0.2–0.89] p = 0.023.

**Conclusions:**

The study found that there was a high incidence of hypotension during elective cesarean section under spinal anesthesia and a significant difference between the SAC and LAC groups. Also, the study found that the independent factors significantly associated with the incidence of hypotension were LAC, higher BMI, higher baby weight, and higher sensory block level, while greater preload volume was protective.

## Introduction

The ideal anesthetic approach for cesarean delivery is regional anesthesia using local anesthetics [1,2]. This is because of the proven reduced morbidity and mortality [3], compared to general anesthesia, which has a higher occurrence of complications, such as difficult intubation, rapid desaturation, a greater chance of aspiration, and neonatal depression. Spinal anesthesia was commonly utilized for cesarean section [4]. However, in parturient, hypotension results from compression of the inferior vena cava, increased abdominal pressure, and decreased lumbosacral subarachnoid space volume [4,5].

Hypotension was the most common complication with an incidence of 15%−30% in the general population with spinal anesthesia [6]. In a cesarean section, hypotension is more concerning [7]. In addition to having a higher incidence (20–80%) in pregnant women, it can have catastrophic effects on the mother and fetus, ranging from an increased occurrence of vomiting and nausea to fetal hypoxia caused by changes in uteroplacental blood flow, which leads to fetal acidosis [7,8].

Post-spinal hypotension was defined as a 20% decrement of MAP (mean arterial blood pressure), clinically more relevant than an absolute value, and it is associated with decreased organ perfusion and adverse maternal and fetal outcomes [9,10]. The effect of abdominal circumference on MAP changes during cesarean section under spinal anesthesia was not fully understood. Some previous studies found that the incidence of hypotension did not differ across the groups (71.42% in the smaller vs. 78.43% in the greater abdominal circumference (AC) group, p = 0.419) [11,12]. However, they indicate that a large abdominal circumference in pregnancy is associated with greater decreases in mean arterial pressure from baseline. Whereas another study reveals that abdominal circumference is a relevant predictor of post-spinal hypotension [13]. The major concern is the lack of standardization in measuring abdominal circumference, which can lead to inconsistent results across studies.

In Ethiopia, there is a high increase in the practice of spinal anesthesia, particularly for C/S [14]. Previous studies in Ethiopia have revealed a significant variability in the incidence of hypotension induced by spinal anesthesia, ranging from 25% to 64% [15,16]. However, the studies didn’t give attention to the association between AC and post-spinal hypotension, and there are conflicting results [11,12].

Despite the previous literature revealing numerous factors associated with the incidence of post-spinal hypotension [10,17–19], it didn’t give attention to the abdominal circumference impact. Furthermore, identifying associated factors that have an impact on the incidence of hypotension is important for the prevention of post-spinal hypotension. In an area with limited resources, prevention methods may yield greater results than treatment protocols. Given this, the study was done to assess the relationship between AC and hypotension during cesarean section under spinal anesthesia. The relationship between abdominal circumference and incidence of hypotension during cesarean section under spinal anesthesia is an area of ongoing research, and understanding this relationship can help in predicting and preventing hypotension. In addition, the study aimed to assess factors associated with the incidence of post-spinal hypotension among mothers undergone elective C/S under spinal anesthesia at a public hospital in Addis Ababa City, Ethiopia.

## Methods and materials

### Study setting

This was conducted in Addis Ababa, the capital city of Ethiopia. The city has a subtropical highland climate. Apart from this, it also hosts foreign embassies and heads of diplomatic missions, international, regional, and sub-regional organizations, and several international NGOs; it is the place of the African Union. Likewise, it has the central station of the United Nations Economic Commission for Africa and various other international organizations. The whole city is divided into 11 sub-cities and 116 woredas with a total area of 520sq.Km. According to a 2017 estimation, Addis Ababa has a population of 6.6 million people. All ethnic groups of the country are represented. In this capital, there are 14 public hospitals, 46 private hospitals, and 86 health centers. The study was conducted in three public hospitals.

First Gandhi Memorial Hospital is the largest maternal and child care center and was established in 1958 by Mohandas Karamchand Gandhi. 4 operating rooms and 120 beds. It serves as a teaching Hospital in collaboration with the Addis Ababa University obstetrics and gynecology department and the College of Health Sciences. Second Menilik II referral hospital provides services with different specialties such as obstetrics and gynecology, medicine, general surgery, cardiothoracic, eyes, orthopedic and trauma, urology, and pediatrics. The hospital offers services with different specialties such as medicine and general surgery. The Third Zewditu Memorial Hospital is located in Kirkos sub city woreda 08, Addis Ababa, Ethiopia. It has 200 beds, and 46 are in the obstetrics, gynecology, and postnatal department. Have 2 elective operative theatres, which were selected for study areas.

### Study design

A multi-center prospective observational study was conducted at public hospitals in Addis Ababa City, Ethiopia, from January 7, 2024, to March 30, 2024.

### Population

The source of the population was all pregnant mothers delivered by cesarean section under spinal anesthesia at public hospitals in Addis Ababa city, Ethiopia, in 2024.

### Study population

Selected pregnant mothers who were delivered by elective cesarean section under spinal anesthesia at public hospitals in Addis Ababa city, Ethiopia, 2024.

### Sample size and sampling technique

The sample size was determined using the single population proportion method for the first objective, which is the incidence of hypotension after spinal anesthesia in term pregnant women, 64% from the previous study [16]. P = 0.64 was used for the calculation to get the maximum sample size, 95% level of significance, 5% margin of error, and 5% for incomplete or contingency data will be used as parameters. These parameters will be used to determine sample size using a single population proportion formula, with assumptions: Z = 1.96, at 95% confidence level; Margin of error = 5%.

Formula: – n= (Z α/2)^2^p (1-p)/ d 2= ((1.96)^2^ *(0.64*0.36))/ (0.05)^2^ = 355. So, n = 355, which is the minimum sample size.

By considering the non-response rate, 5% of the calculated sample size was added, and the final total sample size for the study was 355 + 18 = 373.

### Sampling procedure

After situational analyses were done for the previous three months at each hospital on elective C/S under spinal anesthesia, a random sample of size n is drawn from each hospital. The population of size N is divided into non-overlapping sub-populations of size Gandhi Memorial Hospital (Ng = 368), Minillik 2 Memorial Hospital (Nm = 204), and Zewuditu Memorial Hospital (Nz = 272). The sample was chosen using a method known as systematic random sampling by grouping patients by Hospital. Throughout the study period, a systematic sampling procedure was used to get the required sample size. The first participant was chosen at random, and the remaining participants were chosen based on k = 2. This process was repeated until the desired sample size was reached over the course of the study.

K = N/n, where n = population during the previous three months (846), sampling interval = 846/373 = 2.3 ≈ 2, and total sample size = 373.

Referred to as proportional allocation given by the formula, the sample size in each hospital was established in proportion to the size of the hospital (the number of patients in the hospital) (Fig 1).

**Fig 1:**
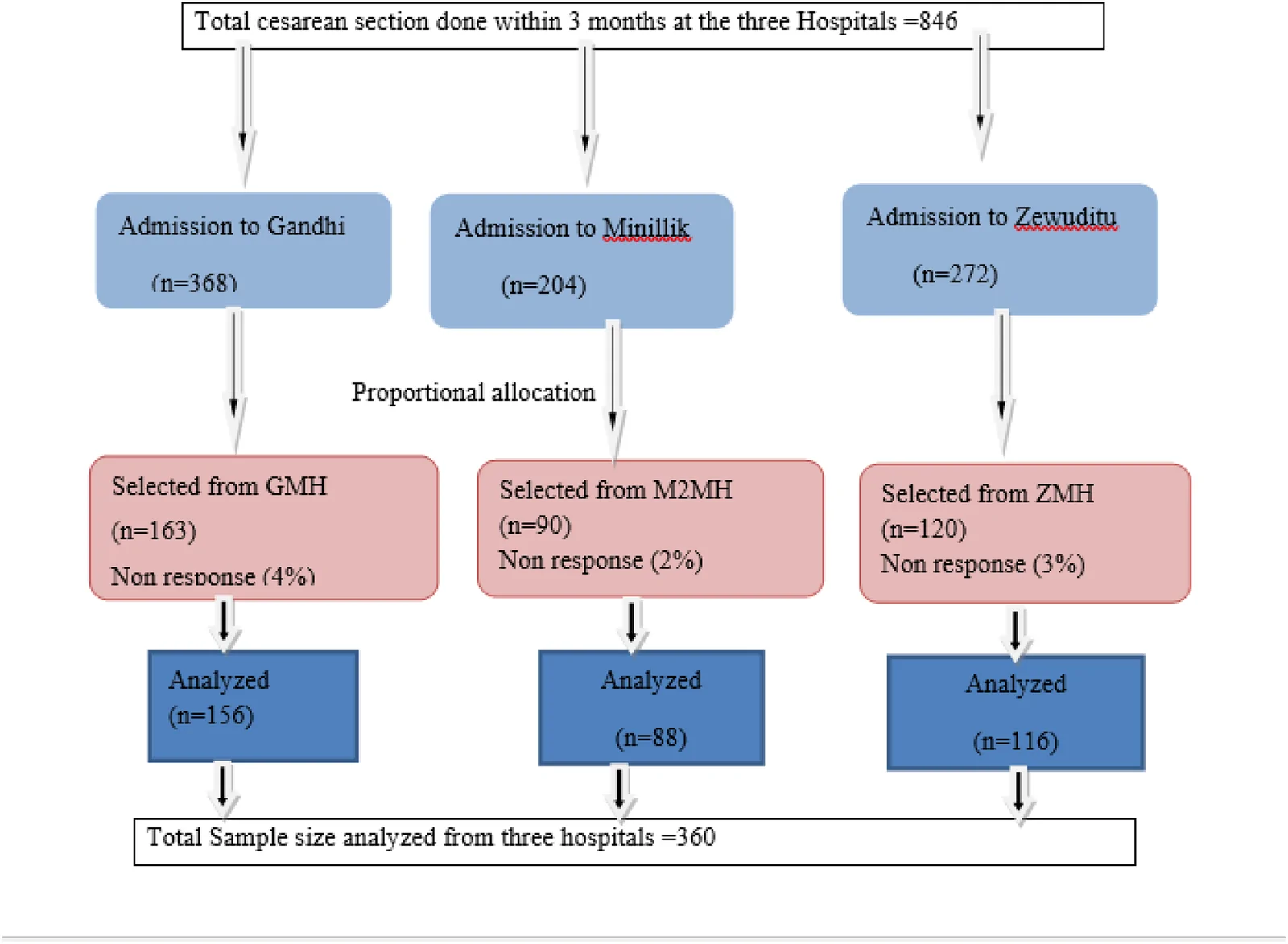
Proportional allocation given by the formula and the sample size in each hospital is established in proportion to the size of the hospital (the number of patients in the hospital).

$n_{i}=\frac{n}{N}*N_{i}$ shown in Fig 1.

ni = proportional allocation

n = total sample size

N = total number of cases found by institutional analysis in 3 hospitals

Ni = total number of cases found by institutional analysis in a single hospital

N = total number of cases found by institutional analysis in 3 hospitals

## Eligibility criteria

### Inclusion criteria

- Pregnant women aged between 15 and 49 years (WHO reproductive age)
- American Society of Anesthesiologists (ASA) class II, III

### Exclusion criteria

- High-risk pregnancy (placenta previa, abruption placentae)
- Eclampsia or pre-eclampsia
- Multiple pregnancies
- Cardiovascular disease/instability, like preoperative hypertension or hypotension
- Prophylactic vasopressor.

### Data collection procedures

A data collection (tool) checklist and questionnaire were developed about pre-anesthesia evaluation sheets, intraoperative monitoring sheets, and other standardized tools from similar studies [11,12,20]. To ensure the quality and consistency of the data, a pretested questionnaire was used to collect data. In addition to sociodemographic factors like age, height, weight, BMI, and ASA status, maternal factors like gravidity, baseline heart rate, baseline blood pressure, and AC, anesthetic and surgical factors like sensory block height, fluid preload, baby weight, estimated blood loss, duration of operation, and intraoperative blood pressure every five minutes were also covered in the questionnaire.

All patients’ abdominal circumferences (AC) were measured in centimeters (cm) at the end of expiration at the umbilical level while they were sitting on the day of operation. Heart rate (beats per minute), systolic blood pressure (mmHg), diastolic blood pressure (mmHg), and mean arterial pressure (mmHg) were among the hemodynamic variables that were measured at baseline.

An attending anesthetist administered spinal anesthesia in the operating room using the standard method. Before the administration of a spinal anesthetic, all patients had routine monitoring using three-lead electrocardiography and pulse oximetry. The patient was put in a sitting position and given 10–12 mg of 0.5% hyperbaric bupivacaine for spinal anesthesia. The Quincke-tip spinal needle gauges 23 and 24 were utilized. Ten minutes after SA induction, the loss of cold feeling to alcohol swabs was used to evaluate the sensory block height. Surgery began after the proper degree of blockage was established. The first patient was recruiting 7/1/ 2024 for the data, and the last patient was recruited on 30/3/2024

### Study variables

- Dependent variable
- Post spinal hypotension (yes/no)
- Independent Variables

### Socio-demographic

- Weight
- Height
- Baby weight
- BMI
- Mother's age

### Obstetric-related factors

- Gravidity
- Baseline HR
- Baseline SB Baseline DBP
- Baseline MAP
- Abdominal circumference

### Anesthesia-related factors

- Pre-load fluid given
- Sensory block height

### Surgical-related factors

- Duration of operation
- Estimated blood loss

### Operational definition

**Abdominal Circumference**: The measurement of the circumference of the abdomen at the level of the umbilicus using a flexible measuring tape (centimeters).

**Hypotension**: In this study, hypotension can be defined as a MAP pressure decrease of 20% or more from baseline or a MAP below 65 mmHg.

**Pregnant Women:** Women who are currently pregnant and scheduled to undergo an elective cesarean section under spinal anesthesia (e.g., 37–42 weeks).

**The degree of sensory block** is indicated by the loss of cold or pinprick feeling, which is bilaterally recorded in the midline clavicle or anterior axillary line.

**Baseline blood pressure** is the measurement made before the administration of spinal anesthesia.

**Preloading**: administering crystalloid fluid before spinal anesthesia induction.

**Large abdominal circumference** ≥ 102 cm.

**Small abdominal circumference** < 102 cm.

### Ethics approval and consent to participate

Ethical approval was obtained from the institutional review board of Addis Ababa University and was brought to the office of the hospital. Then, a formal letter of permission to conduct the study was obtained. A legal letter was also submitted to Gandhi Memorial Hospital, Minilik Referral Hospital, and Zewuditu Memorial Hospital, where the study took place. Oral informed consent was also obtained from each study participant after a clear orientation of the study objective, benefits, and procedures. Confidentiality of participants’ information was maintained using unique codes and medical record numbers rather than personal identification. Moreover, the data collected from each study participant were used solely for the intended purpose.

### Data processing and analysis

The Patients were categorized by their median abdominal circumference, < 102 cm (small abdominal circumference (SAC)) and ≥102 cm (large abdominal circumference (LAC). The incidence of post-spinal hypotension was estimated with a 95% confidence interval. Pearson's chi-squared test was performed to compare the LAC and SAC groups. Multicollinearity was also examined using the Variance Inflation Factor (VIF), and it was determined that no multicollinearity existed. The Hosmer-Lemeshow test was utilized to evaluate model fitness. The test result indicates that the model fits the data well (Hosmer–Lemeshow test, p = 0.63), suggesting no significant difference between the observed and predicted outcomes.

Bivariate binomial logistic regression was done to evaluate the relationship between a single independent and dependent variable. All variables with a p-value ≤0.25 in bivariate logistic regression were entered into multivariable regression to control for all possible confounders, and the variables were selected by the backward stepwise method to see the effect of each variable on the outcome variables. Finally, the findings of the multivariable logistic regression analysis were presented using the adjusted odds ratio along with their 95% confidence interval. The level of statistical significance was declared at a p-value ≤0.05.

### Data quality management

To ensure data quality, two days of training were given to data collectors on the aim of the study and the content of the data abstraction format. One week before actual data collection started, pre-testing was done at Yekatit 12 Hospital on 10% of the sample size to assess the clarity, sequence, validity, consistency, and understandability of the data collection tools, and a few modifications were made.

The suitability and validity of the data collection format were also assessed through discussion with the anesthesia professionals. The principal investigator and supervisor supervised the data collection process daily to ensure that the data were accurate, consistent, and comprehensive. The missing data for each patient were checked daily by supervisors who acted accordingly.

## Results

**Socio-demographic and clinical characteristics of obstetric patients undergoing elective cesarean section under spinal anesthesia at public hospitals in Addis Ababa city, Ethiopia, 2024.**

A total of 360 were involved in the analysis and allocated into two groups by their median abdominal circumference (102 cm). All of the study participants responded to the interview for socio-demographic characteristics and were observed for clinical characteristics. The majority of the patients (60%) were between 25 and 35 years old, 50.6% (91) for the SAC and 70% (126) for the LAC group. Among all the sampled participants, 38.33% had a BMI between 25–29.9 kg/m2, which is 29.2% (54) for SAC and 84(48.0%) for the LAC group; whereas 16.95% (61) of patients had BMI ≥ 30 kg/m2, which is 10.8% (20) for SAC and 23.4% (41) for the LAC groups. The majority (95.3%) of study subjects were ASA II, which is 98.3% (177) and 92.2% (166) for SAC and LAC groups, respectively. Generally, two-thirds of the pregnant women were gravida one or two, which is 72.78% among SAC and 59.44% among LAC groups (Table 1).

**Table 1:** Descriptive data on the distribution of socio-demographic and clinical characteristics with abdominal circumference status of obstetric patients undergoing elective cesarean section under spinal anesthesia at public hospitals of Addis Ababa, Ethiopia, 2024.

Variables	Categories	Frequency (%)	
		SAC (n = 180)	LAC (n = 180)
Age of the parturient	15-24	63(35.0%)	22(12.2%)
	25-35	91(50.6%)	126(70.0%)
	36-49	26(14.4%)	32(17.8%)
	Total	180(100%)	180(100%)
BMI of the parturient	18.5-4.9 kg/m2	111(60.0%)	50(28.6%)
	25-29.9 kg/m2	54(29.2%)	84(48.0%)
	>/ = 30kgm2	20(10.8%)	41(23.4%)
	Total	180(100%)	180(100%)
ASA status	ASA class 2	177(98.3%)	166(92.2%)
	ASA class 3	3(1.7%)	14(7.8)
	Total	180(100%)	180(100%)
Gravida of the patient	Gravida 1	88(48.9%)	51(28.3%)
	Gravida 2	43(23.9%)	56(31.1%)
	Gravida 3	39(21.7%)	59(32.8%)
	Gravida 4	9(5.0%)	12(6.7%)
	Gravida above 4	1(0.6%)	2(1.1%)
	Total	180(100%)	180(100%)
Level sensory of block	T4	5(2.7%)	5(2.9%)
	T6	77(42.7%)	112(62.3%)
	T8	18(10%)	5(2.9%)
	T10	80(44.3%)	58(32.0%)
	Total	180(100%)	180(100%)
Fluid given in ml	<500	43(23.9%)	44(24.4%)
	500-1000	137(76.1%)	136(75.6%)
	Total	180(100%)	180(100%)
Duration of operation in minutes	Mean ±SD	36.36 ± 10.426	36.39 ± 8.274
Blood loss estimation in ml	500-1000	174(96.7%)	171(95%)
	>1000	6(3.3%)	9(5%)
	Total	180(100%)	180(100%)
Baby's weight in kg	<2.5	32(17.3%)	9(5.1%)
	2.5-4	149(80.5%)	139(79.4%)
	>4	4(2.2%)	27(15.4%)
	Total	180(100%)	180(100%)
Baseline HR (bpm)		83.96 ± 13.71	87.24 ± 12.75
Baseline MAP (mmHg)		79.75 ± 9.45	82.64 ± 10.65

Most of the sensory block level for the SAC group was T10, which is 44.3% (82), whereas T6 among the LAC groups which 62.3% (109). The majority of the patients were preloaded with a range of 500–1000 ml fluid for both groups, 76.1% (137%) and 75.6% (136) among SAC and LAC groups, respectively. The average baseline SBP of both groups was 121.29 ± 12.59 and 126.98 ± 11.81S in the SAC and LAC group, respectively, as well as average baseline DBP of both groups was 75.42 ± 11.77 and 77.30 ± 10.95 mmHg in the SAC and LAC group, respectively. Among sampled patients, the mean of baseline HR was 83.96 ± 13.71 and 87.24 ± 12.75 in the SAC and LAC group, respectively. Among the study participants, the mean of baseline MAP was 79.75 ± 9.45 and 82.64 ± 10.65 in the SAC and LAC groups, respectively.

In this study, the majority of the babies’ weight was classified as 2.5 kg to 4 kg for both groups, 149 (80.5%) for the SAC group and 139(79.4%) for the LAC. Vasopressor was given to a few patients, SAC 6 (3.33%) and LAC 15 (8.3%) (Table 1).

**The cumulative Incidence of hypotension among the mothers who underwent elective cesarean section under spinal anesthesia at the public hospitals in Addis Ababa City, Addis Ababa, Ethiopia,** 2024**.**

The cumulative incidence of post-spinal hypotension was calculated with nighty five percent confidence intervals. In this study, the cumulative incidence of post-spinal hypotension was 76.67% with 95%CI [71.9–80.9] from a total of 360 pregnant mothers, as shown in Fig 2.

**Fig 2:**
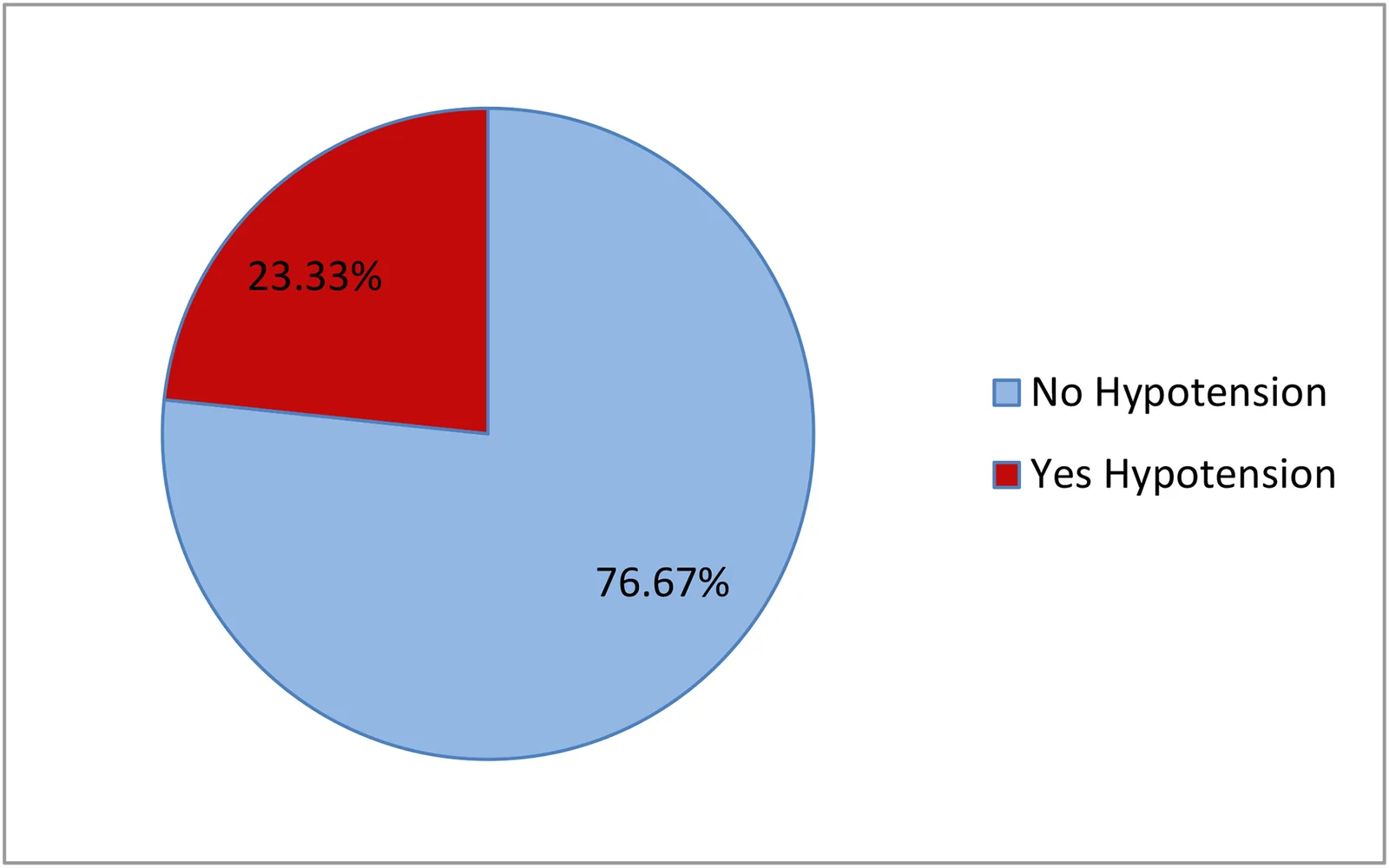
Incidence of post-spinal hypotension among obstetric patients undergoing elective cesarean sectionThe relationship between the abdominal circumference and incidence of hypotension among mothers who underwent elective cesarean section under spinal anesthesia at public hospitals in Addis Ababa City, Addis Ababa, Ethiopia, 2024.

The incidence between groups was 63.3% in the smaller AC group vs. 85.6% in the larger AC group, p < 0.001. There was strong evidence of a relationship between AC and incidence of hypotension (Chi-square = 51.3, df = 1 and p < 0.001). There was a statistically significant relationship between AC and the incidence of hypotension. There was a significant association between LAC and post-spinal hypotension (AOR = 2.98, 95% CI [1.57, 5.65]). The other predictors that were independently associated with post-spinal hypotension were BMI, baby weight, and sensory block height. In the larger AC group, there was a greater maximal fall in MAP following spinal anesthesia from baseline (Table 2).

**Table 2:** Hemodynamic outcomes in the study group among obstetric patients undergoing elective cesarean section under spinal anesthesia at public hospitals of Addis Ababa, Ethiopia, 2024.

	SAC (180)	LAC (180)	P VALUE
The incidence of hypotension	123(63.3%)	154(85.6%)	<0.001
Maximal MAP percentage decrease from baseline (percent)	28.62 ± 12.86	35.28 ± 11.65	<0.001
Vasopressors given	6(3.3%)	15(8.3%)	0.49

The incidence of significant hypotension, which is defined as a decrease in MAP of more than 20% from the baseline, was higher in the larger AC group than in the smaller group, as indicated by the change in MAP percentage according to the study groups shown in Table 2 and Fig 3 (63.3% in the smaller AC group vs. 85.6% in the larger AC group, p < 0.001).

**Fig 3:**
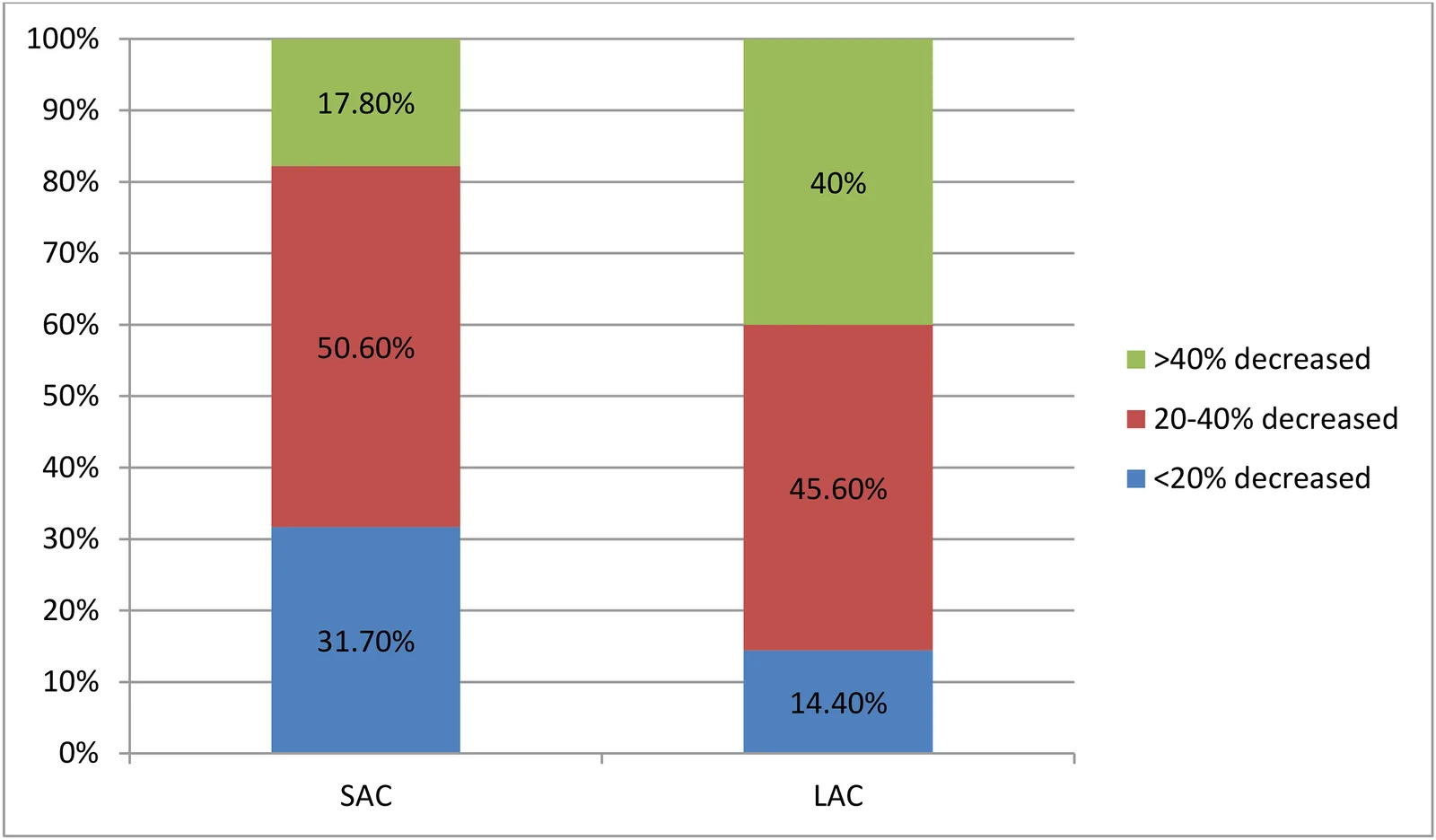
Stratification for each group based on the percentage drop in mean arterial pressure from baseline among obstetric patients undergoing elective cesarean section under spinal anesthesia at public hospitals of Addis Ababa, Ethiopia, 2024.

The data were presented as percentages stratified by each study group's mean arterial pressure drop from baseline as a percentage. The percentage decrement of MAP from baseline to 20–40% in the SAC group was 50.6%, whereas the percentage decrement of MAP from baseline to 20–40% in the SAC group was 45.6%. MAP decreased from baseline to >40% in the SAC group by 17.8%, whereas in the LAC group decreased by 40%; p value < 0.05 Table 2.

Factors associated with post-spinal hypotension among the mothers who underwent elective cesarean section under spinal anesthesia at public hospitals in Addis Ababa city, Ethiopia, 2024

Bivariate logistic regression was done to evaluate the association between a single independent and dependent variable. The correlation between those independent variables was checked for the multi-collinearity effect by using the variance inflation factor (VIF) and tolerance test for those p-values <0.25. To account for all potential confounders, multivariable regression was used for all variables with p-values ≤0.25 in bivariate regression. In bivariate analysis, AC, BMI, level of sensory block, baby weight, and duration of operation, preload fluid, ASA, age, baseline MAP, and weight were candidates for multivariate regression (Table 3). Three of the factors that were statistically significant in bivariate regression, including ASA, age, and baseline MAP, did not show a statistically significant association with the post-spinal anesthesia hypotension in multivariate logistic regression. Multivariate logistic regression analysis revealed a statistically significant association between post-spinal hypotension and the patient's BMI, AC, degree of sensory block, weight, preload fluid, and infant weight (P-value ≤0.05) (Table 3).

**Table 3:** Bivariate and Multivariate logistic regression model to identify factors associated with the incidence of post spinal hypotension among obstetric patients undergoing elective cesarean section under spinal anesthesia at public hospitals of Addis Ababa, Ethiopia, 2024.

Variables	Category	Hypotension status of pregnant		COR, 95% CI	AOR, 95% CI	P value
		<20%/no	>20%/yes			
Level of sensory block	T10	42(30.4%)	96(69.6%)	1	1	
	T8	8(33.3%)	16(66.7%)	3.93(0.48-32.08) *	8.07(0.9-72.39)	0.062
	T6	33(17.6%)	155(82.4%)	2.05(1.22-3.46) *	2.22(1.12-4.42)	0.023
	T4	1(10.0%)	9(90.0%)	0.87(0.35-2.2)	2.18(0.75-6.35)	0.15
Weight in kg	Mean ±SD	66.33 ± 11.45	69.31 ± 12.23	1.02(1.00-1.04) *	0.96(0.92-0.98)	0.006
BMI in kg/m2	18.5-24.9	38(24.7)	116(75.3%)	1	1	
	25-29.9	43(26.5%)	119(73.5%)	0.14(0.03-0.63) *	2.3(1.16-4.6)	0.018
	≥30	2(4.5%)	42(95.5%)	0.13(0.03-0.57) *	3.19(1.09-9.07)	0.034
Abdominal circumference	SAC	57(31.7%)	123(68.3%)	1	1	
	LAC	26(14.4%)	154(85.6%)	0.364(0.27-0.61) *	2.98(1.57-5.65)	0.001
Fluid given preoperatively in ml	<500	12(13.8%	75(86.2%)	1	1	
	500-1000	71(26.0%)	202(74.0%)	2.2(1.13-4.28) *	0.42(0.2-0.89)	0.023
Duration of operation	Mean ±SD	34.52 ± 5.61	36.93 ± 10.2	1.04(1.00-1.08) *	1.08(1.03-1.13)	0.002
Baby weight	<2.5	14(42.4%)	19(57.6%)	1	1	
	2.5-4	67(22.6%)	229(77.4%)	0.094(0.02-0.46) *	4.08(1.79-9.25)	0.001
	>4	2(6.5%)	29(93.5%)	0.25(0.06-1.01) *	11.41(2.0-64.86)	0.006

*- > a candidate variable in binary regression @ p<=0.25. ** = indicates statistically significant in multivariable logistic regression @p<=0.05.

LAC was associated with a three times higher likelihood of having hypotension (AOR = 2.98, 95% CI [1.57, 5.65]). Obese patients (>30 kg/m2) were more than three times more likely to have hypotension (AOR = 3.19, 95%CI [1.09, 9.07]). As the weight of the patient increased by one kg, the incidence of hypotension was 0.96 times less likely to have hypotension (AOR 0.96, 95% CI, [0.92–0.98]). The odds of having hypotension were more than eleven times higher among patients who had > 4 kg babies compared to patients with < 2.5 kg baby weight (AOR 11.41, 95%CI, [2.0, 64.86]).

The odds of having hypotension were two times higher among patients who had sensory height block above T6 level when compared to sensory block at T10 (AOR 2.22, 95%CI [1.12, 4.42]). Patients with pre-operative 500−1000 ml fluid loading were 0. 42 times less likely to have hypotension compared to patients with 500 ml pre-loading (AOR 0.42, 95% CI, [0.2–0.89]). The duration of operation, as increased by one-minute incidence of hypotension increased by two approximately (AOR 1.08, 95% CI, [1.03–1.13]).

## Discussion

The primary finding of this study was 76.67% (95%CI [71.9–80.9]) incidence of post-spinal hypotension among elective mothers delivered with C/S. The result is comparable with the study done in Thailand [12] and less than the study in Ethiopia, which revealed the incidence of post-spinal hypotension was 80% in five to fifteen minutes and 83.7% in fifteen to twenty-five minutes [15]. In contrast, the result was greater than the previous report in Ethiopia, which was 64% the differences could be due to the difference in the definition given for post-spinal hypotension they used [16]. Another research done in Ethiopia assessed the incidence of hypotension without considering the impact of AC on the incidence of hypotension, reported 25% [17]. This result was higher than studies done in Rwanda (40%), Brazil (33%), Germany (56%), and Thailand (65.1% [7,10,21,22].. The discrepancy could be due to the definition given for post-spinal hypotension, which is defined as an SBP decrease of 30% from baseline in most previous studies [22] and a decrease of 20% from baseline in the current study.

The current study also identifies that there was a relationship between the decrease in MAP and AC, which is proven by previous studies [20,23]. In the same way, the bigger AC group saw a greater maximal fall in MAP following spinal anesthesia from baseline, and the MAP percentage decrement of mean was 28.62 ± 12.86 in SAC and 35.38 ± 11.65 in LAC, with a p-value of less than 0.001, which is comparable with previous studies [12,20]. The incidence of significant hypotension was higher in the larger AC group than in the smaller group (63.3% in the smaller AC group vs. 85.6% in the larger AC group, p < 0.001), in contrast to previous studies [13]. The large gravid uterus in a term parturient might result in aorto-caval compression, which lowers cardiac output and venous return when the patient is supine. The size of the uterus is reflected in the measurement of abdominal circumference during pregnancy; therefore, the higher the AC, the greater the fall in MAP. The abdominal circumference and abdominal pressure have a positive correlation. It's been demonstrated that high spinal anesthesia and hypotension can result from elevated abdominal pressure [19,24].

In our study, when other co-factors were controlled, LAC was associated with a three times higher likelihood of having hypotension (AOR = 2.98, 95% CI [1.57, 5.65]) compared to SAC. This result is comparable with the prior studies [23]. This implies that post-spinal hypotension care and intervention strategies relating to elective cesarean section for term pregnant women with large abdominal circumference should be strengthened. So, it is recommended to include an abdominal circumference parameter to assist anesthetists in preparing for hypotension events after spinal anesthesia in pregnant women.

Furthermore, the current study discovered that several factors are associated with post-spinal hypotension. In the current study, the higher levels of sensory blockage were statistically significant in association with the incidence of hypotension, which is similar to a previous study [25–27]. This could be the result of sympathectomy. Additionally, because of increased IVC compression and thus greater epidural venous plexus distension, parturients with higher AC had lower volumes of lumbosacral CSF [11]. Our results show that there is a two-fold increase in the likelihood of developing hypotension for sensory height blocks above T6, with an odds ratio of (AOR = 2.22 (95% CI: 1.12–4.42)). These results are in line with those who discovered a relationship between a sensory block height above T6 and an increased risk of hypotension [7,28,29].

The current finding shows that a BMI of > 25 kg/m2 was three times more likely to develop hypotension than a patient with a lower BMI. This result is consistent with previous findings that the incidence of hypotension was higher in over weight than in the normal groups [7,30]. However, our result did not correlate with a previous study that found BMI had no significant association with incidence of hypotension, an odds ratio of 1.07 (0.74–1.75) with a p value of 0.591 [20].

Our findings support those of the previous reports that demonstrated baby weight had a strong association with the incidence of hypotension. In this study, babies with a weight of>=4 kg were eleven times more likely to develop hypotension. This finding is similar to prior studies, which identified that the weight of the newborn plays a crucial role in the occurrence of hypotension after spinal anesthesia during cesarean section. Specifically, newborns with a weight of ≥4 kg were found to be significantly associated with a higher risk of developing hypotension compared to newborns with a weight of ≤2.4 kg [16,31]. According to this, there is a greater chance of hypotension with increasing infant weight because of things like the gravid uterus compressing the major arteries and inferior vena cava, which lowers venous return and causes hypotension [12].

The findings in the previous study pointed out that a 11–20 kg weight gain during pregnancy above the antenatal weight was a risk factor, with a 5-fold increase in the risk of developing severe hypotension. However, the study didn’t assess the association between the weight of parturients and the incidence of Hypotension. Our current study confirms that the association between parturient weight and the incidence of post-spinal hypotension [18].

The other confounding factor, the amount of fluid given preoperatively, also has an association with hypotension during cesarean section under spinal anesthesia in the current study, which shares previous study results [15,17]. Another study noted that the incidence of post-spinal hypotension was reduced in patients who received crystalloid 20 ml/kg than in patients without pre-hydration [32]

### Strengths and limitations of the study

The study's strengths were being a multi-center study and a large sample size compared to previous studies.

There are certain limitations to our study as well. Firstly, since the goal of our study was to maintain the level of spinal anesthesia at T4, we did not look at the impact of the degree of spinal anesthesia with big AC. In addition, since the main intention of this study is to assess the relationship between AC and post-spinal hypotension, we did not measure abdominal pressure that could be affected by conditions such as oligohydramnios, polyhydramnios, or central obesity. Future studies with larger sample sizes and more detailed clinical measurements are recommended to better evaluate their independent effects. Moreover, we did not follow up with those patients who had developed hypotension over an extended period to identify any further problems or to ascertain the patient's fate.

## Conclusion

The incidence of hypotension was high, and significant association between LAC and the incidence of post-spinal hypotension. As well as the incidence between the groups was different. MAP of LAC significantly decreased from base line than MAP among the SAC group. BMI > 30 kg/m2, baby weight > 4 kg, large parturient weight, and sensory block height above T6 were significantly associated with post-spinal hypotension during elective cesarean section.

### Recommendation

First, measuring abdominal circumference as part of the preoperative evaluation to identify patients at high risk of developing hypotension is recommended for anesthesia professionals. Secondly, we recommend that anesthesia providers increase monitoring of blood pressure and other vital signs in patients with large abdominal circumferences to identify early signs of hypotension and consider using fluid administration, blood pressure support, or vasopressor.

Moreover, we recommend conducting further studies on this topic, including exploring other factors like the ratio of trunk length and abdominal circumference, and direct abdominal pressure that may influence this relationship.

## Supporting information

S1 File wajjeee44. (CSV)

## Abbreviations

AC: abdominal circumference
ASA: American society of anesthesiology
BMI: body mass index
CI: confidence interval
C/S: cesarean section
CSF: cerebrospinal fluid
DBP: diastolic blood pressure
IVC: inferior vena cava compression
LAC: large abdominal circumference
MAP: mean arterial pressure
SA: spinal anesthesia
SAC: small abdominal circumference
SBP: systolic blood pressure
