# Supplementary material for: Abdominal circumference and its relationship with the incidence of hypotension during elective cesarean section under spinal anesthesia at public hospitals of Addis Ababa, Ethiopia, 2024
Source: PLoS One. 2026 Oct 23;21(10):e0354843. doi: 10.1371/journal.pone.0354843 (PMC13693878; doi:10.1371/journal.pone.0354843)
Supplement: Supplementary file 1 [file pone.0354843.s002.docx]

This is a comprehensive and clinically relevant research manuscript investigating an important issue in obstetric anesthesia. Below is a detailed, section-by-section critique covering strengths, weaknesses, and suggestions for improvement.

**Overall Strengths**

1. Relevant Topic: The study addresses a common and serious complication (hypotension) in a specific, high-volume clinical setting (elective cesarean section under spinal anesthesia).
2. Clear Objectives: The primary and secondary aims are well-defined.
3. Prospective Design: A multi-center prospective observational design is appropriate for establishing incidence and identifying associations.
4. Adequate Sample Size: The sample size (n=360) is substantial and appears well-calculated for the primary objective.
5. Practical Implications: The findings have direct clinical applicability for risk stratification and management in resource-limited settings.
6. Ethical Compliance: The study received appropriate ethical approvals and consent.

**Detailed Comments & Critiques**

**1. Title & Abstract**

- **Suggestions:**

**Title:** Consider specifying "term pregnant women" to match inclusion criteria.

**Methods:** Briefly state the cutoff for LAC/SAC (102 cm) here, as it's a key variable.

**Results:** The sentence "Large abdominal circumference in pregnancy is associated with greater decreases in mean arterial pressure from baseline" is a conclusion. Rephrase to present the finding, e.g., "The LAC group showed a greater maximal decrease in MAP from baseline compared to the SAC group (35.28% vs. 28.62%, p<0.001)."

**Conclusions:** It states "the independent factors... were LAC, BMI, baby weight, amount of preload given, and sensory block." However, in the results, "amount of preload given" (AOR=0.42) is a protective factor. Clarify: "...were LAC, higher BMI, higher baby weight, and higher sensory block level, while greater preload volume was protective."

**2. Introduction**

Good background on the significance of post-spinal hypotension and the rationale for studying abdominal circumference (AC). Correctly identifies the gap in knowledge and standardization.

Question

- **Literature Gap:** The argument could be sharper. Instead of saying previous studies "didn’t give attention," highlight the conflicting results from cited studies (e.g., Anadani et al. 2021 vs. Thomard et al. 2020) and how your study, with a larger sample and standardized measurement, aims to clarify this.
- **Flow:** Paragraphs could be more streamlined. The transition from global/Ethiopian context to the specific study aim is slightly abrupt.

**3. Methods**

Detailed description of setting, sampling (proportional allocation is a strength), data collection, and operational definitions.

- **Major Questions:**
- **Definition of Hypotension:** The study uses "a MAP decrease of ≥20% from baseline **or** a MAP <65 mmHg." This is problematic.
- **Clinical Relevance:** In obstetric anesthesia, hypotension is most commonly defined as a **percentage drop in Systolic Blood Pressure (SBP)** (e.g., >20-30% from baseline) or an absolute SBP <90-100 mmHg. MAP is less commonly used as the sole criterion in this context.
- **"Or" creates ambiguity:** A patient with a baseline MAP of 100 dropping to 81 (19% drop, MAP >65) would not be hypotensive. A patient with a baseline MAP of 70 dropping to 66 (5.7% drop, MAP <65) would be hypotensive. This captures very different physiological states. **Justify this definition robustly with references, or consider aligning with more standard obstetric definitions (SBP-based).**
- **Sample Size Justification:** The calculation is based on a 64% incidence from a prior study. It's acceptable but could be strengthened by also performing a sample size calculation for the comparison between SAC and LAC groups (the primary relationship), which might require a different n.
- **Exclusion Criteria:** Excluding patients receiving "prophylactic vasopressor" is standard but should be explicitly defined (e.g., "receiving any vasopressor infusion or bolus prior to the onset of hypotension").
- **Measurement of AC:** "At the umbilical level while sitting" is good. Was this done at end-expiration? This should be standardized and stated.
- **Spinal Anesthesia Technique:** The dose of hyperbaric bupivacaine is **not mentioned**. This is a **critical omission** as drug dose is a major determinant of block height and hypotension risk. State the standard dose used (e.g., 10-12 mg).
- **Confounding Variables:** Good list. Was **baseline heart rate** considered as a potential confounder? Tachycardia might indicate reduced venous return.

**4. Results**

Clear tables and figures. Well-presented demographic and clinical characteristics.

Questions

- **Table 1 & Data Presentation:**
  1. The "Level sensory of block" row in Table 1 has "Total" placed incorrectly; it should be under the "Variables" column.
  2. For continuous variables like Baseline HR/MAP, presenting Mean ±SD for the entire cohort and by hypotension status (Yes/No) would be more informative than just by SAC/LAC.
  3. **Statistical Analysis Concerns:**
- **Categorization of Continuous Variables:** BMI, baby weight, and preload fluid were categorized for analysis. While common, this loses information. Consider reporting results from analyses treating these as continuous variables (e.g., per 5 kg/m² increase in BMI, per 0.5 kg increase in baby weight) in addition to or instead of categories, especially for the multivariate model.
- **Odds Ratio Interpretation:** The interpretation of the weight variable is confusing: "As the weight of the patient increased by one kg the incidence of hypotension was 0.96 times less likely" (AOR 0.96, 95% CI, [0.92-0.98]). **An AOR of 0.96 for a 1 kg increase suggests a very small protective effect,** which seems counterintuitive given that weight and BMI are correlated, and higher BMI is a risk factor. This warrants explanation in the discussion—could it be residual confounding, or does maternal weight independently have a different relationship when BMI is in the model?
- **Model Fit and Variable Selection:** Mentioning the Hosmer-Lemeshow test and backward selection is good. Report the p-value for the Hosmer-Lemeshow test to confirm "good fit" (p > 0.05).

4. **Figure 3:** The caption and text reference "SAC group" twice in error. Correct the legend/description.

**5. Discussion**

Attempts to contextualize findings within existing literature, explaining discrepancies (e.g., definition of hypotension). Good pathophysiological reasoning for the AC-hypotension link.

Questions

1. **Overstatement of Novelty:** While important, the finding that LAC is associated with hypotension has been reported previously (as cited). The discussion should more humbly position this as a **confirmation and quantification** of that relationship in a large Ethiopian cohort, highlighting the high incidence found.
2. **Inconsistent Comparisons:** When comparing incidence rates with other studies (e.g., Rwanda 40%, Brazil 33%), the discussion correctly attributes differences to varying definitions. This point is well-made.
3. **Unexplained Findings:** The counterintuitive result for **maternal weight** (AOR 0.96) is not addressed. This needs an explanation—could it be collinearity with BMI? Was variance inflation factor (VIF) checked for multicollinearity between weight, BMI, and AC?
4. **Mechanism:** The discussion on mechanisms (aorto-caval compression, CSF volume) is good but could be more precise. Cite studies that directly link AC or fundal height to reduced CSF volume or increased epidural pressure.
5. **Strength of Association:** For baby weight >4kg (AOR=11.4), the confidence interval is **extremely wide** [2.0-64.86], indicating great uncertainty in this estimate due to a small number of macrosomic babies (n=31 total, with 29 in hypotension group). Highlight this as a **preliminary finding requiring validation in a cohort with more macrosomic cases.**

**6. Limitations**

- The stated limitations are appropriate (no abdominal pressure measurement, no long-term follow-up). Why?

**7. Formatting & Minor Issues**

- **References:** Some references in the text (e.g., (11, 12, 20) in Methods) are incomplete; ensure all are listed in the reference section.
- **Typos/Grammar:** There are minor errors throughout (e.g., "night five percent" -> "ninety-five percent", "vasopressor, SAC 6(33.3%)" likely means "SAC 6(3.3%)"). A careful proofread is needed.
- **Data Availability Statement:** The statement on pages 5-6 is cut off and contradictory. It must be completed clearly, following PLOS ONE policy (e.g., "Data are available upon reasonable request due to ethical restrictions concerning patient confidentiality. Requests can be made to the Institutional Review Board of Addis Ababa University at [contact email].").

**Overall Summary and Priority Revisions**

This is a valuable study with a strong foundation. To strengthen it for publication, the authors should prioritize:

1. Clarify and Justify the Primary Outcome: Defend the MAP-based definition or switch to an SBP-based definition standard in obstetrics.
2. Report the Spinal Anesthetic Dose: This is a critical methodological detail.
3. Address Statistical Anomalies: Explain the counterintuitive AOR for maternal weight and acknowledge the imprecise estimate for macrosomia.
4. Sharpen the Discussion: Focus on confirming and quantifying known relationships in this specific population, and explain discrepancies in results more thoroughly.
5. Complete and Polish: Fix formatting errors, complete the data availability statement, and perform a thorough language edit.
