# Supplementary material for: Abdominal circumference and its relationship with the incidence of hypotension during elective cesarean section under spinal anesthesia at public hospitals of Addis Ababa, Ethiopia, 2024
Source: PLoS One. 2026 Oct 23;21(10):e0354843. doi: 10.1371/journal.pone.0354843 (PMC13693878; doi:10.1371/journal.pone.0354843)
Supplement: Peer review history

# Peer review: Decision Letter 0

**DOI:** 10.1371/journal.pone.0354843.r001

**Authors:** Alexander Wolf

3 Feb 2026

Dear Dr. Kono,

Thank you for submitting your manuscript to PLOS ONE. After careful consideration, we feel that it has merit but does not fully meet PLOS ONE’s publication criteria as it currently stands. Therefore, we invite you to submit a revised version of the manuscript that addresses the points raised during the review process.

==============================

Please address all the points made by the reviewers und clarify the issues regarding methodology and statistics.

==============================

Please submit your revised manuscript by Mar 20 2026 11:59PM. If you will need more time than this to complete your revisions, please reply to this message or contact the journal office at plosone@plos.org. When you're ready to submit your revision, log on to https://www.editorialmanager.com/pone/ and select the 'Submissions Needing Revision' folder to locate your manuscript file.

- A letter that responds to each point raised by the academic editor and reviewer(s). You should upload this letter as a separate file labeled 'Response to Reviewers'.
- A marked-up copy of your manuscript that highlights changes made to the original version. You should upload this as a separate file labeled 'Revised Manuscript with Track Changes'.
- An unmarked version of your revised paper without tracked changes. You should upload this as a separate file labeled 'Manuscript'.

If you would like to make changes to your financial disclosure, please include your updated statement in your cover letter. Guidelines for resubmitting your figure files are available below the reviewer comments at the end of this letter.

If applicable, we recommend that you deposit your laboratory protocols in protocols.io to enhance the reproducibility of your results. Protocols.io assigns your protocol its own identifier (DOI) so that it can be cited independently in the future. For instructions see: https://journals.plos.org/plosone/s/submission-guidelines#loc-laboratory-protocols. Additionally, PLOS ONE offers an option for publishing peer-reviewed Lab Protocol articles, which describe protocols hosted on protocols.io. Read more information on sharing protocols at https://plos.org/protocols?utm_medium=editorial-email&utm_source=authorletters&utm_campaign=protocols.

We look forward to receiving your revised manuscript.

Kind regards,

Alexander Wolf

Academic Editor

PLOS One

Journal Requirements:

When submitting your revision, we need you to address these additional requirements.

1. Please ensure that your manuscript meets PLOS ONE's style requirements, including those for file naming. The PLOS ONE style templates can be found at

https://journals.plos.org/plosone/s/file?id=wjVg/PLOSOne_formatting_sample_main_body.pdf and

https://journals.plos.org/plosone/s/file?id=ba62/PLOSOne_formatting_sample_title_authors_affiliations.pdf

2. Thank you for stating the following in your Competing Interests section:

“The authors declare they have no competing interests.”

Please complete your Competing Interests on the online submission form to state any Competing Interests. If you have no competing interests, please state "The authors have declared that no competing interests exist.", as detailed online in our guide for authors at http://journals.plos.org/plosone/s/submit-now

This information should be included in your cover letter; we will change the online submission form on your behalf.

3. We note that you have indicated that there are restrictions to data sharing for this study. For studies involving human research participant data or other sensitive data, we encourage authors to share de-identified or anonymized data. However, when data cannot be publicly shared for ethical reasons, we allow authors to make their data sets available upon request. For information on unacceptable data access restrictions, please see http://journals.plos.org/plosone/s/data-availability#loc-unacceptable-data-access-restrictions.

Before we proceed with your manuscript, please address the following prompts:

a) If there are ethical or legal restrictions on sharing a de-identified data set, please explain them in detail (e.g., data contain potentially identifying or sensitive patient information, data are owned by a third-party organization, etc.) and who has imposed them (e.g., a Research Ethics Committee or Institutional Review Board, etc.). Please also provide contact information for a data access committee, ethics committee, or other institutional body to which data requests may be sent.

b) If there are no restrictions, please upload the minimal anonymized data set necessary to replicate your study findings to a stable, public repository and provide us with the relevant URLs, DOIs, or accession numbers. Please see http://www.bmj.com/content/340/bmj.c181.long for guidelines on how to de-identify and prepare clinical data for publication. For a list of recommended repositories, please see https://journals.plos.org/plosone/s/recommended-repositories. You also have the option of uploading the data as Supporting Information files, but we would recommend depositing data directly to a data repository if possible.

Please update your Data Availability statement in the submission form accordingly.

4. Your ethics statement should only appear in the Methods section of your manuscript. If your ethics statement is written in any section besides the Methods, please move it to the Methods section and delete it from any other section. Please ensure that your ethics statement is included in your manuscript, as the ethics statement entered into the online submission form will not be published alongside your manuscript.

5. Please include captions for your Supporting Information files at the end of your manuscript, and update any in-text citations to match accordingly. Please see our Supporting Information guidelines for more information: http://journals.plos.org/plosone/s/supporting-information.

6. If the reviewer comments include a recommendation to cite specific previously published works, please review and evaluate these publications to determine whether they are relevant and should be cited. There is no requirement to cite these works unless the editor has indicated otherwise.

[Note: HTML markup is below. Please do not edit.]

Reviewers' comments:

Reviewer's Responses to Questions

**Comments to the Author**

1. Is the manuscript technically sound, and do the data support the conclusions?

Reviewer #1: Partly

Reviewer #2: Yes

Reviewer #3: Yes

Reviewer #4: Yes

**********

2. Has the statistical analysis been performed appropriately and rigorously?

Reviewer #1: Yes

Reviewer #2: Yes

Reviewer #3: Yes

Reviewer #4: Yes

**********

3. Have the authors made all data underlying the findings in their manuscript fully available?

The PLOS Data policy

Reviewer #1: Yes

Reviewer #2: Yes

Reviewer #3: Yes

Reviewer #4: Yes

**********

4. Is the manuscript presented in an intelligible fashion and written in standard English?

Reviewer #1: Yes

Reviewer #2: Yes

Reviewer #3: No

Reviewer #4: Yes

**********

Reviewer #1: This is a valuable study with a strong foundation. To strengthen it for publication, the authors should prioritize:

1. Clarify and Justify the Primary Outcome: Defend the MAP-based definition or switch to an SBP-based definition standard in obstetrics.

2. Report the Spinal Anesthetic Dose: This is a critical methodological detail.

3. Address Statistical Anomalies: Explain the counterintuitive AOR for maternal weight and acknowledge the imprecise estimate for macrosomia.

4. Sharpen the Discussion: Focus on confirming and quantifying known relationships in this specific population, and explain discrepancies in results more thoroughly.

5. Complete and Polish: Fix formatting errors, complete the data availability statement, and perform a thorough language edit.

Reviewer #2: In the study population, the authors stated that "Selected pregnant mothers who were delivered by elective cesarean section under spinal anesthesia at public hospitals in Addis Ababa city, Ethiopia, 2024" How did you select these patients from the source population? Please provide your clarification.

In your eligibility criteria, does having oligohydramnios or polyhydramnios or having obesity, especially central obesity, or fetal macrosomia affect your dependent variable? Please provide your justification for this, or add this to the limitation section.

Have you included or excluded the parturients who are hypotensive preoperatively? If yes, does it have any impact on your dependent variable? If not, include it in an exclusion criterion.

The authors stated that 500-1000 ml of fluid was administered to the patients. Here, which patient groups took 500 ml, and which patient group took 1000 ml? The amount of fluid administered has an impact on the incidence of hypotension. So please provide your justification for this. It is better if you make it protocol-based (i.e., 10 ml/kg or 15 ml/kg).

How were you checked for the normality of the data?

How were you analyzed for non-parametric variables?

Please mention if there is any dropout or missed patient during your analysis.

The majority of the patients in the LAC groups have higher dermatomal blockage in comparison to the SAC group in your study; that means this patient group probably has a high incidence of hypotension due to sympathectomy. Do you think this may directly affect your outcome variable? If yes, how and what method have you tried to control this? Please provide a justification. or consider this in your limitation section.

Reviewer #3: Authors have chosen a very thoughtful topic. The study plan has been very comprehensive and integrating multiple study sites with organized manner is well appreciated. However, there are several corrections needed which are as follows:

1. The researchers wanted to assess the relationship between the abdominal circumference of pregnant women and the occurrence of hypotension. My query is that why did you want to assess abdominal circumference? What should be done with the present research's findings in a preventive manner (mention in the introduction).

2. Mention ASA's and VIF abbreviation in text first, then continue as it is.

3. Add units of variables (such as SPB, etc.). Also, recheck the units intext.

4. Line 241-43, grammatical errors.

Reviewer #4: First, I would like to congratulate the authors on conducting a study aimed at improving care for an important patient population.

I have the following questions and comments for clarification and discussion:

Abdominal circumference cutoff

Please clarify how the cutoff value of 102 cm was determined to define small versus large abdominal circumference. Was this value derived from prior literature, population percentiles, ROC curve analysis, or another methodological approach?

Abstract clarification

In the Abstract (line 25), it is not clear whether receiving a higher volume of fluids is identified as a protective factor or a risk factor. Based on the statistical analysis, it appears to be a protective factor; however, this should be explicitly stated to avoid ambiguity.

Novelty and clinical applicability

Although a different cutoff for abdominal circumference is proposed, the overall finding aligns with existing knowledge—namely, that patients with higher BMI, larger fetuses, and increased abdominal circumference are more likely to experience hypotension following spinal anesthesia.

From a clinical perspective, it would be helpful for the authors to elaborate on how this specific measurement would alter anesthesia practice. For example:

Would this influence the choice of anesthetic technique?

Would it prompt adjustment of the spinal anesthetic dose?

Would it change perioperative fluid or vasopressor management?

Additionally, while lower spinal doses may result in higher block levels in this population, such blocks may regress more rapidly, potentially leading to insufficient duration of anesthesia in patients with high BMI. Addressing this trade-off would strengthen the clinical relevance of the findings.

Implications for resource-limited settings

Finally, the Introduction suggests that this approach may help reduce costs in low-resource settings. It would be valuable to clarify how the use of abdominal circumference measurement would translate into cost reduction, particularly in terms of anesthesia management, complication prevention, or resource utilization.

**********

what does this mean?). If published, this will include your full peer review and any attached files.

If you choose “no”, your identity will remain anonymous but your review may still be made public.

**Do you want your identity to be public for this peer review?**  For information about this choice, including consent withdrawal, please see our Privacy Policy

Reviewer #1: **Yes:**  Atalay Eshetie Demilie

Reviewer #2: No

Reviewer #3: **Yes:**  Tabeer Tanwir Awan

Reviewer #4: No

**********

[NOTE: If reviewer comments were submitted as an attachment file, they will be attached to this email and accessible via the submission site. Please log into your account, locate the manuscript record, and check for the action link "View Attachments". If this link does not appear, there are no attachment files.]

To ensure your figures meet our technical requirements, please review our figure guidelines: https://journals.plos.org/plosone/s/figures

You may also use PLOS’s free figure tool, NAAS, to help you prepare publication quality figures: https://journals.plos.org/plosone/s/figures#loc-tools-for-figure-preparation.

NAAS will assess whether your figures meet our technical requirements by comparing each figure against our figure specifications.

Attachment Submitted filename: PLOS ONE.docx

# Author comment: Author response to Decision Letter 1

**DOI:** 10.1371/journal.pone.0354843.r002

13 May 2026

A Point -by-point response to reviewer comments

We would like to thank the editor and the reviewer for their thoughtful comments and constructive suggestions. We appreciate the time and effort they have dedicated to reviewing our manuscript. We have carefully considered all the feedback and have made revisions to address the concerns raised. However, we are happy to make further adjustments if the reviewer feels additional correction is necessary.

Reviewer 1

1. “Clarify and Justify the Primary Outcome: Defend the MAP-based definition or switch to an SBP-based definition standard in obstetrics.”

We thank the reviewers for pointing out this important comment. MAP was selected because it reflects the average perfusion pressure of vital organs and is commonly used to assess tissue perfusion during anesthesia. In obstetric anesthesia, maintaining adequate maternal organ and uteroplacental perfusion is critical, and MAP provides a more comprehensive measure of circulatory status than a single systolic blood pressure value.

Although systolic blood pressure (SBP) based definitions are widely used in obstetric literature, particularly in studies of spinal anesthesia for cesarean delivery, MAP has increasing support in perioperative research as a physiologically meaningful indicator of perfusion. Several anesthesia studies have used MAP thresholds to define hypotension because it better represents the pressure driving blood flow to essential organs.

Therefore, in this study, MAP was used as the primary measure to identify hypotension during anesthesia. However, to maintain consistency with obstetric anesthesia literature, SBP values were also monitored and recorded throughout the procedure

2. “Report the Spinal Anesthetic Dose: This is a critical methodological detail.”

Thank you for this valuable suggestion. We have reviewed and incorporated the issues.

3. “Address Statistical Anomalies: Explain the counterintuitive AOR for maternal weight and acknowledge the imprecise estimate for macrosomia.”

Thank you for highlighting the importance of cautious interpretation of statistically estimates of maternal anthropometric and fetal factors in predicting hypotension. After thoroughly reviewing the manuscript, we have rechecked the Multicollinearity using the VIF, and it was determined that no multicollinearity exist among raised factors. Therefore, the observed association was not reflected residual confounding or interaction with other variables included in the model.

4. “Sharpen the Discussion: Focus on confirming and quantifying known relationships in this specific population, and explain discrepancies in results more thoroughly.”

We appreciate the reviewer’s insightful comment regarding sharpening the Discussion we have tried to sharpen the discussion part

4.1. Unexplained Findings: The counterintuitive result for maternal weight (AOR 0.96) is not addressed. This needs an explanation—could it be collinearity with BMI? Was variance inflation factor (VIF) checked for multicollinearity between weight, BMI, and AC?

We have rechecked the Multicollinearity using the VIF, and it was determined that no multicollinearity exist among raised factors

4.2. “Strength of Association: For baby weight >4kg (AOR=11.4), the confidence interval is extremely wide [2.0-64.86], indicating great uncertainty in this estimate due to a small number of macrosomia babies (n=31 total, with 29 in hypotension group).”

Thank you for this important observation. Regarding fetal macrosomia, the wide confidence interval suggests an imprecise estimate, most likely due to the relatively small number of macrosomia cases in the sample. We have acknowledged this limitation in the revised manuscript and emphasized that the result should be interpreted with caution.

5. “Complete and Polish: Fix formatting errors, complete the data availability statement, and perform a thorough language edit.

Thank you for this helpful comment. We carefully reviewed the entire manuscript and corrected formatting inconsistencies throughout the document. The Data Availability Statement has now been completed and added to the manuscript to clearly describe how the study data can be accessed. In addition, the manuscript has undergone a thorough language revision to improve clarity, grammar, and overall readability. We believe these changes have significantly improved the overall quality and presentation of the manuscript

5.1. “References: Some references in the text (e.g., (11, 12, 20) in Methods) are incomplete; ensure all are listed in the reference section”.

After carefully reviewing the paper and given comments, we tried to react and incorporate them.

5.2. “Typos/Grammar: There are minor errors throughout (e.g., "night five percent" -> "ninety-five percent", "vasopressor, SAC 6(33.3%)" likely means "SAC 6(3.3%)"). A careful proofread is needed.”

We genuinely appreciate the reviewer’s thoughtful recommendation. After careful reviewing we identify typos/grammar error and we tried to correct it.

5.3 “Model Fit and Variable Selection: Mentioning the Hosmer-Lemeshow test and backward selection is good. Report the p-value for the Hosmer-Lemeshow test to confirm "good fit" (p > 0.05).”

Thank you for the comment. We have now reported the p-value of the Hosmer–Lemeshow goodness-of-fit test in the results section to assess the adequacy of the logistic regression model. The test result indicates that the model fits the data well (Hosmer–Lemeshow test, p > 0.63), suggesting no significant difference between the observed and predicted outcomes.

Reviewer #2

Point -by-point response to reviewer comments

1. “In the study population, the authors stated that "Selected pregnant mothers who were delivered by elective cesarean section under spinal anesthesia at public hospitals in Addis Ababa city, Ethiopia, 2024" How did you select these patients from the source population?”

We would like to thank the editor and the reviewer for their thoughtful comments and constructive suggestions. We have explained detail in the method section. The sample was chosen using a systematic random sampling by grouping patients by Hospital. Throughout the study period, a systematic sampling procedure was used to get the required sample size. The first participant was chosen at random, and the remaining participants were chosen based on k=2, this process was repeated until the desired sample size was reached over the course of the study.

2. “In your eligibility criteria, does having oligohydramnios or polyhydramnios or having obesity, especially central obesity, or fetal macrosomia affect your dependent variable? Please provide your justification for this, or add this to the limitation section.”

Thank you for this important comment. Conditions such as oligohydramnios, polyhydramnios, maternal obesity, particularly central obesity, and fetal macrosomia may influence intra-abdominal pressure and aortocaval compression, which can potentially affect maternal hemodynamic changes and the incidence of hypotension during spinal anesthesia. However, the primary focus of this study was to examine the relationship between abdominal circumference and the occurrence of hypotension during elective cesarean section under spinal anesthesia. Abdominal circumference was used as a proxy measure that may partly reflect these physiological and anatomical variations.

In our dataset, detailed clinical stratification for conditions such as oligohydramnios, polyhydramnios, or central obesity was limited, and therefore these variables were not included as separate exclusion criteria or covariates in the final analysis. We have now acknowledged this in the limitation section of the manuscript, noting that these conditions may act as potential confounders and could influence the observed association. Future studies with larger sample sizes and more detailed clinical measurements are recommended to better evaluate their independent effects.

3. “Have you included or excluded the parturient who are hypotensive preoperatively?”

We appreciate this important comment! Cardiovascular instability/disease like preoperative hypertension or hypotension was excluded.

4. “The authors stated that 500-1000 ml of fluid was administered to the patients. Here, which Patient groups took 500 ml, and which patient group took 1000 ml? The amount of fluid administered has an impact on the incidence of hypotension. So please provide your justification for this. It is better if you make it protocol-based (i.e., 10 ml/kg or 15 ml/kg)”.

Thank you for this valuable comment. In our study setting, preloading with crystalloid solution was routinely administered prior to spinal anesthesia to reduce the risk of spinal-induced hypotension. The volume of fluid administered ranged from ‘500 to 1000 ml”, depending on the patient’s body weight, hemodynamic status, and the anesthetist’s clinical judgment at the time of the procedure. Patients with relatively lower body weight generally received approximately 500 ml, while those with higher body weight or greater estimated intravascular volume requirement received closer to 1000 ml. We agree that a weight-based protocol such as 10–15 ml/kg provides better standardization. However, due to variations in clinical practice among anesthetists in the participating hospitals, a strictly protocol-based fluid regimen was not consistently applied during the study period. Saying this the amount of preoperative fluid given was one of factor associated with dependent factor.

5. “How were you checked for the normality of the data? How were you analyzed for non-parametric variables?”

We appreciate the reviewer’s attention to detail. The normality of continuous variables was assessed using both statistical tests and graphical methods. The Shapiro–Wilk test was used to statistically evaluate the normality of the data distribution. In addition, histograms and Q–Q plots were examined to visually assess whether the data followed a normal distribution. The data was normal distributed!

6. Please mention if there is any dropout or missed patient during your analysis.

No dropout/missed

7. “The majority of the patients in the LAC groups have higher dermatomal blockage in comparison to the SAC group in your study; that means this patient group probably has a high incidence of hypotension due to sympathectomy. Do you think this may directly affect your outcome variable? If yes, how and what method have you tried to control this? Please provide a justification. or consider this in your limitation section”

We thank the reviewer for pointing out this important point. The correlation between those independent variables was checked for multi-collinearity effect, by using variance inflation factor (VIF) and tolerance test for those p values <0.25. We have rechecked the Multicollinearity using the VIF, and it was determined that no multicollinearity exist among raised factors.

Point -by-point response to reviewer comments

Reviewer 3

We would like to thank the editor and the reviewer for their thoughtful comments and constructive suggestions. We appreciate the time and effort they have dedicated to reviewing our manuscript. We have carefully considered all the feedback and have made revisions to address the concerns raised. However, we are happy to make further adjustments if the reviewer feels additional correction is necessary.

1. “The researchers wanted to assess the relationship between the abdominal circumference of pregnant women and the occurrence of hypotension. My query is that why did you want to assess abdominal circumference? What should be done with the present research's findings in a preventive manner (mention in the introduction).”

We have thoroughly reviewed the introduction and we have incorporated it.

2. Mention ASA's and VIF abbreviation in text first, then continue as it is.

We appreciate this comment. We have reviewed and reacted accordingly.

3. Add units of variables (such as SPB, etc.). Also, recheck the units intext.

We appreciate this comment. We have reviewed and reacted accordingly.

4. Line 241-43, grammatical errors

Thank you for this comment and we have reacted accordingly

Reviewer #4:

1. “Abdominal circumference cutoff. Please clarify how the cutoff value of 102 cm was determined to define small versus large abdominal circumference. Was this value derived from prior literature, population percentiles, ROC curve analysis, or another methodological approach?”

We thank the reviewer for pointing out this important point. Most of previous study were used median Abdominal circumference as cutoff point. Similarly, in this study the patients were categorized by their median abdominal circumference, <102cm (small abdominal circumference (SAC)) and ≥102cm (large abdominal circumference (LAC). Most of previous study were used median Abdominal circumference.

2. “Abstract clarification. In the Abstract (line 25), it is not clear whether receiving a higher volume of fluids is identified as a protective factor or a risk factor. Based on the statistical analysis, it appears to be a protective factor; however, this should be explicitly stated to avoid ambiguity”

We appreciate this comment. This is the editorial issues and we tried to correct it. We agree that the mentioned factor is protective!

3. “Although a different cutoff for abdominal circumference is proposed, the overall finding aligns with existing knowledge—namely, that patients with higher BMI, larger fetuses, and increased abdominal circumference are more likely to experience hypotension following spinal anesthesia.

From a clinical perspective, it would be helpful for the authors to elaborate on how this specific measurement would alter anesthesia practice. For example:

Would this influence the choice of anesthetic technique?

Would it prompt adjustment of the spinal anesthetic dose?

Would it change perioperative fluid or vasopressor management?

Additionally, while lower spinal doses may result in higher block levels in this population, such blocks may regress more rapidly, potentially leading to insufficient duration of anesthesia in patients with high BMI. Addressing this trade-off would strengthen the clinical relevance of the findings.”

Thank you for the important comment and points! This study is a single study and it give some recommendation but it is difficult to change the doses of drugs and clinical management based on single study results.

4. “Implications for resource-limited settings

Finally, the Introduction suggests that this approach may help reduce costs in low-resource settings. It would be valuable to clarify how the use of abdominal circumference measurement would translate into cost reduction, particularly in terms of anesthesia management, complication prevention, or resource utilization”

In clinical and practical implications this study couldn’t decrease cost directly but we hope avoiding complication like post spinal anesthesia could facilitate early recovery and discharge.

Once again, we thank the editors and reviewers for their valuable feedback. We believe that the revisions have significantly improved the manuscript, and we hope it now meets the journal’s standards for publication. We look forward to hearing from you.

# Peer review: Decision Letter 1

**DOI:** 10.1371/journal.pone.0354843.r003

**Authors:** Alexander Wolf

25 Jun 2026

Dear Dr. Kono,

Thank you for submitting your manuscript to PLOS ONE. After careful consideration, we feel that it has merit but does not fully meet PLOS ONE’s publication criteria as it currently stands. Therefore, we invite you to submit a revised version of the manuscript that addresses the points raised during the review process.

==============================

Please submit your revised manuscript by Aug 09 2026 11:59PM. If you will need more time than this to complete your revisions, please reply to this message or contact the journal office at plosone@plos.org. When you're ready to submit your revision, log on to https://www.editorialmanager.com/pone/ and select the 'Submissions Needing Revision' folder to locate your manuscript file.

- A letter that responds to each point raised by the academic editor and reviewer(s). You should upload this letter as a separate file labeled 'Response to Reviewers'.
- A marked-up copy of your manuscript that highlights changes made to the original version. You should upload this as a separate file labeled 'Revised Manuscript with Track Changes'.
- An unmarked version of your revised paper without tracked changes. You should upload this as a separate file labeled 'Manuscript'.

If you would like to make changes to your financial disclosure, please include your updated statement in your cover letter. Guidelines for resubmitting your figure files are available below the reviewer comments at the end of this letter.

If applicable, we recommend that you deposit your laboratory protocols in protocols.io to enhance the reproducibility of your results. Protocols.io assigns your protocol its own identifier (DOI) so that it can be cited independently in the future. For instructions see: https://journals.plos.org/plosone/s/submission-guidelines#loc-laboratory-protocols. Additionally, PLOS ONE offers an option for publishing peer-reviewed Lab Protocol articles, which describe protocols hosted on protocols.io. Read more information on sharing protocols at https://plos.org/protocols?utm_medium=editorial-email&utm_source=authorletters&utm_campaign=protocols.

As the corresponding author, your ORCID iD is verified in the submission system and will appear in the published article. PLOS supports the use of ORCID, and we encourage all coauthors to register for an ORCID iD and use it as well. Please encourage your coauthors to verify their ORCID iD within the submission system before final acceptance, as unverified ORCID iDs will not appear in the published article. *Only*  the individual author can complete the verification step; PLOS staff *cannot*  verify ORCID iDs on behalf of authors.

We look forward to receiving your revised manuscript.

Kind regards,

Alexander Wolf

Academic Editor

PLOS One

Journal Requirements:

If the reviewer comments include a recommendation to cite specific previously published works, please review and evaluate these publications to determine whether they are relevant and should be cited. There is no requirement to cite these works unless the editor has indicated otherwise.

Please review your reference list to ensure that it is complete and correct. If you have cited papers that have been retracted, please include the rationale for doing so in the manuscript text, or remove these references and replace them with relevant current references. Any changes to the reference list should be mentioned in the rebuttal letter that accompanies your revised manuscript. If you need to cite a retracted article, indicate the article’s retracted status in the References list and also include a citation and full reference for the retraction notice.

[Note: HTML markup is below. Please do not edit.]

Reviewers' comments:

Reviewer's Responses to Questions

**Comments to the Author**

Reviewer #2: All comments have been addressed

Reviewer #4: All comments have been addressed

**********

2. Is the manuscript technically sound, and do the data support the conclusions?

Reviewer #2: Yes

Reviewer #4: Yes

**********

3. Has the statistical analysis been performed appropriately and rigorously?

Reviewer #2: Yes

Reviewer #4: Yes

**********

4. Have the authors made all data underlying the findings in their manuscript fully available?

The PLOS Data policy

Reviewer #2: Yes

Reviewer #4: Yes

**********

5. Is the manuscript presented in an intelligible fashion and written in standard English?

Reviewer #2: Yes

Reviewer #4: Yes

**********

Reviewer #2: The overall document is well-written; however, some grammatical errors need correction. Please correct it.

Reviewer #4: (No Response)

**********

what does this mean?). If published, this will include your full peer review and any attached files.

If you choose “no”, your identity will remain anonymous but your review may still be made public.

**Do you want your identity to be public for this peer review?**  For information about this choice, including consent withdrawal, please see our Privacy Policy

Reviewer #2: **Yes:**  Mesay Milkias

Reviewer #4: No

**********

[NOTE: If reviewer comments were submitted as an attachment file, they will be attached to this email and accessible via the submission site. Please log into your account, locate the manuscript record, and check for the action link "View Attachments". If this link does not appear, there are no attachment files.]

To ensure your figures meet our technical requirements, please review our figure guidelines: https://journals.plos.org/plosone/s/figures

You may also use PLOS’s free figure tool, NAAS, to help you prepare publication quality figures: https://journals.plos.org/plosone/s/figures#loc-tools-for-figure-preparation.

NAAS will assess whether your figures meet our technical requirements by comparing each figure against our figure specifications.

# Author comment: Author response to Decision Letter 2

**DOI:** 10.1371/journal.pone.0354843.r004

4 Jul 2026

We sincerely thank the reviewer for this positive and constructive comment. In response, we have carefully revised the entire manuscript to improve its grammar, language, clarity, and readability. We believe that the revisions have significantly improved the manuscript, and we hope it now meets the journal’s standards for publication. We look forward to hearing from you.

# Editor report: Decision Letter 2

**DOI:** 10.1371/journal.pone.0354843.r005

**Authors:** Alexander Wolf

13 Jul 2026

Abdominal circumference and its relationship with the incidence of hypotension during elective cesarean section under spinal anesthesia at public hospitals of Addis Ababa, Ethiopia, 2024.

PONE-D-25-65624R2

Dear Dr. Kono,

We’re pleased to inform you that your manuscript has been judged scientifically suitable for publication and will be formally accepted for publication once it meets all outstanding technical requirements.

Within one week, you’ll receive an e-mail detailing the required amendments. When these have been addressed, you’ll receive a formal acceptance letter and your manuscript will be scheduled for publication.

An invoice will be generated when your article is formally accepted. Please note, if your institution has a publishing partnership with PLOS and your article meets the relevant criteria, all or part of your publication costs will be covered. Please make sure your user information is up-to-date by logging into Editorial Manager at Editorial Manager® and clicking the ‘Update My Information' link at the top of the page. For questions related to billing, please contact billing support.

If your institution or institutions have a press office, please notify them about your upcoming paper to help maximize its impact. If they’ll be preparing press materials, please inform our press team as soon as possible -- no later than 48 hours after receiving the formal acceptance. Your manuscript will remain under strict press embargo until 2 pm Eastern Time on the date of publication. For more information, please contact onepress@plos.org.

Kind regards,

Alexander Wolf

Academic Editor

PLOS One

Additional Editor Comments (optional):

Reviewers' comments:

# Editor report: Acceptance letter

**DOI:** 10.1371/journal.pone.0354843.r006

**Authors:** Alexander Wolf

PONE-D-25-65624R2

PLOS One

Dear Dr. Kono,

I'm pleased to inform you that your manuscript has been deemed suitable for publication in PLOS One. Congratulations! Your manuscript is now being handed over to our production team.

At this stage, our production department will prepare your paper for publication. This includes ensuring the following:

* All references, tables, and figures are properly cited

* All relevant supporting information is included in the manuscript submission,

* There are no issues that prevent the paper from being properly typeset

You will receive further instructions from the production team, including instructions on how to review your proof when it is ready. Please keep in mind that we are working through a large volume of accepted articles, so please give us a few days to review your paper and let you know the next and final steps.

Lastly, if your institution or institutions have a press office, please let them know about your upcoming paper now to help maximize its impact. If they'll be preparing press materials, please inform our press team within the next 48 hours. Your manuscript will remain under strict press embargo until 2 pm Eastern Time on the date of publication. For more information, please contact onepress@plos.org.

You will receive an invoice from PLOS for your publication fee after your manuscript has reached the completed accept phase. If you receive an email requesting payment before acceptance or for any other service, this may be a phishing scheme. Learn how to identify phishing emails and protect your accounts at https://explore.plos.org/phishing.

If we can help with anything else, please email us at customercare@plos.org.

Thank you for submitting your work to PLOS One and supporting open access.

Kind regards,

PLOS One Editorial Office Staff

on behalf of

Dr. Alexander Wolf

Academic Editor

PLOS One
